# An Update on the Reversal of Non-Vitamin K Antagonist Oral Anticoagulants

**DOI:** 10.1155/2020/7636104

**Published:** 2020-01-27

**Authors:** Mark Terence P. Mujer, Manoj P. Rai, Varunsiri Atti, Ian Limuel Dimaandal, Abigail S. Chan, Shiva Shrotriya, Krishna Gundabolu, Prajwal Dhakal

**Affiliations:** ^1^Department of Medicine, Michigan State University, East Lansing, MI, USA; ^2^Department of Internal Medicine, University of Connecticut, Farmington, CT, USA; ^3^Department of Internal Medicine, Sinai Hospital of Baltimore, Baltimore, MD, USA; ^4^Division of Oncology and Hematology, Department of Internal Medicine, University of Nebraska Medical Center, Omaha, NE, USA; ^5^Fred and Pamela Buffett Cancer Center, University of Nebraska Medical Center, Omaha, NE, USA

## Abstract

Non-vitamin K antagonist oral anticoagulants (NOACs) include thrombin inhibitor dabigatran and coagulation factor Xa inhibitors rivaroxaban, apixaban, edoxaban, and betrixaban. NOACs have several benefits over warfarin, including faster time to the achieve effect, rapid onset of action, fewer documented food and drug interactions, lack of need for routine INR monitoring, and improved patient satisfaction. Local hemostatic measures, supportive care, and withholding the next NOAC dose are usually sufficient to achieve hemostasis among patients presenting with minor bleeding. The administration of reversal agents should be considered in patients on NOAC's with major bleeding manifestations (life-threatening bleeding, or major uncontrolled bleeding), or those who require rapid anticoagulant reversal for an emergent surgical procedure. The Food and Drug Administration (FDA) has approved two reversal agents for NOACs: idarucizumab for dabigatran and andexanet alfa for apixaban and rivaroxaban. The American College of Cardiology (ACC), American Heart Association (AHA), and Heart Rhythm Society (HRS) have released an updated guideline for the management of patients with atrial fibrillation that provides indications for the use of these reversal agents. In addition, the final results of the ANNEXA-4 study that evaluated the efficacy and safety of andexanet alfa were recently published. Several agents are in different phases of clinical trials, and among them, ciraparantag has shown promising results. However, their higher cost and limited availability remains a concern. Here, we provide a brief review of the available reversal agents for NOACs (nonspecific and specific), recent updates on reversal strategies, lab parameters (including point-of-care tests), NOAC resumption, and agents in development.

## 1. Introduction

**Figure 1:**
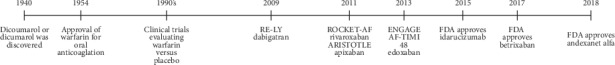
Oral anticoagulants and NOAC reversal agents' timeline.

Non-vitamin K antagonist oral anticoagulants (NOACs) have become the cornerstone in the prevention and treatment of venous thromboembolism (VTE) in nonvalvular atrial fibrillation. For years, vitamin K antagonists (VKA) and heparin derivatives were the only available anticoagulants. From 1954 until the advent of non-vitamin K antagonist oral anticoagulants (NOACs) in 2010, warfarin was the only available oral agent (see Figure 1).

RE‐LY trial compared Dabigatran, which is the first developed NOAC with warfarin in patients with nonvalvular atrial fibrillation. The higher 150 mg dose was associated with a lower rate of stroke and systemic embolism (SE) but a similar rate in major bleeding compared to warfarin. A lower 110 mg dose was similar to warfarin in the prevention of stroke and SE and was associated with a lower rate of major bleeding. Patients with age <75 years were reported to have a lower rate of major bleeding and major extracranial bleeding compared to warfarin for both doses of dabigatran [1]. The results from the ROCKET-AF trial showed rivaroxaban to be noninferior to warfarin for the prevention of stroke or SE [2]. Rivaroxaban was associated with less frequent intracranial and fatal bleeding, but there was no significant group difference in the risk of major bleeding. The ARISTOTLE trial found that apixaban was superior to warfarin in preventing stroke or SE. Also, it was associated with a lower rate of major bleeding and lower mortality [3]. The ENGAGE AF-TIMI 48 showed that once-daily edoxaban (either 30 mg or 60 mg) was non-inferior to warfarin in the prevention of stroke or systemic embolism. Edoxaban was associated with a dose-dependent decrease in the rate of major bleeding, intracranial bleeding, and life-threatening bleeding. However, a higher dose of edoxaban caused a higher rate of gastrointestinal bleeding compared to warfarin [4].

For the treatment of acute VTE, six clinical trials have compared dabigatran, rivaroxaban, apixaban, and edoxaban with conventional therapy (parenteral anticoagulation followed by VKA) [5]. In the dabigatran and the edoxaban trials, patients in both the NOAC and conventional therapy arm received 5 days of parenteral anticoagulation before starting either dabigatran or edoxaban. However, in the rivaroxaban and the apixaban trials, the agents were initiated without prior parenteral anticoagulation. The primary efficacy outcomes for all four NOACs were non-inferior to conventional treatment—dabigatran (HR 1.09; 95% CI: 0.76 to 1.57) [6, 7], rivaroxaban (HR: 0.89; 95% CI: 0.66 to 1.19) [8], apixaban (relative risk (RR): 0.84; 95% CI: 0.60 to 1.18) [9], and edoxaban (HR: 0.89; 95% CI: 0.70 to 1.13) [6] in the referenced phase III clinical trials. Apixaban was associated with a significant reduction in major bleeding compared with conventional treatment (RR: 0.31; 95% CI: 0.17 to 0.55) [9]. The outcome was similar for rivaroxaban in the pulmonary embolism study but not in the deep vein thrombosis (DVT) trial [10]. Edoxaban, dabigatran and rivaroxaban were safer than conventional treatment with lower clinically relevant bleeding (HR: 0.81; 95% CI: 0.71 to 0.94) [11], and (HR: 0.62; 95% CI: 0.50 to 0.76) [7], and (HR: 0.93; 95% CI: 0.81 to 1.06) respectively [8]. The reduction in intracranial hemorrhage with all NOACs was statistically non-significant, and not adequately sized to show a definitive effect. There was a significant reduction in fatal bleeding in the Hokusai study which compared edoxaban with warfarin (2 events vs. 10 events; odds ratio (OR): 0.20; 95% CI: 0.04 to 0.91) [11]. Unfortunately, there is no definitive data available on the incidence of gastrointestinal or mucosal bleeding with NOACs. Twice-daily apixaban could be preferred for its safety profile, as shown by the 69% reduction in major bleeding [9], also since United States Food and Drug Administration (FDA) recently approved generics of apixaban making it more affordable. Betrixaban, which is the fifth oral, direct factor Xa inhibitor, has been approved by the FDA for VTE prophylaxis in acutely ill medical patients. However, betrixaban has not been studied in acute VTE or in the prevention of VTE recurrence.

NOACs have several advantages over warfarin including faster time to achieve the anticoagulant effect, a shorter plasma half-life, lack of need for routine INR monitoring, and improved patient satisfaction [12]. There have been concerns about achieving hemostasis among patients on NOACs with bleeding episodes since there were no specific reversal agents available until 2015. Two reversal agents were recently approved by FDA, the American College of Cardiology (ACC), American Heart Association (AHA), and Heart Rhythm Society (HRS) (ACC/AHA/HRS) released guidelines on their appropriate use. We mainly discuss the management of bleeding episodes with NOACs, including the use of nonspecific and specific reversal agents, application of lab parameters, and resumption of NOAC. Also, we briefly touch base upon agents in development.

## 2. Initiation of NOAC

Prior to the initiation of NOACs, comorbidities and comedications (with emphasis on P-glycoprotein and CYP3A4 interacting medicines) should be carefully reviewed [13–15]. Baseline laboratory workup, namely, hemoglobin, liver, and renal function as well as a coagulation panel should be ordered to assess if a patient is an appropriate candidate for the initiation of NOAC or if dosing adjustment is needed. Older age with increased fall risk, previous major bleed, ongoing heavy alcohol use, and coronary artery disease requiring percutaneous coronary intervention are concerning factors for initiation of NOACs. The data on drug-drug interactions in patients on NOACs is limited [13]. Based on the available data, concurrent use of amiodarone, fluconazole, rifampin, or phenytoin with NOACs has been reported to be associated with an increased predisposition for major bleeding [13]. Therefore, physicians should use caution when prescribing the above medications in patients on NOAC. Additionlly, the International Society of Thrombosis and Haemostasis does not support the use of NOACs in patients with a BMI of >40 kg/m^2^ or weight of >120 kg due to concern for decreased drug exposure and risk of underdosing [16].

## 3. Indications for Reversal Agents

The most recent American Heart Association/American College of Cardiology/Heart Rhythm Society (ACC/AHA/HRS) guidelines define major bleeding as all major bleeds that are associated with either hemodynamic compromise, bleed in a critical organ site (e.g., intracranial and pericardial), a drop in hemoglobin >2 g/dL (when baseline is unknown), or a need for 2 units of whole blood or red cells [17]. Patients presenting with life-threatening bleeding, major uncontrolled bleeding, or requiring rapid anticoagulant reversal for emergent surgical procedures are candidates for the use of reversal agents. In patients with NOAC-related intracerebral hemorrhage (NOAC-ICH), immediate administration of reversal agent is recommended to prevent life-threatening bleeding complications [18].

Nonmajor bleed/minor bleed is any bleed that does not meet the criteria for major bleed. In cases of minor bleed, local hemostatic measures, supportive care, and withholding the next doses of the NOAC are usually sufficient to control the bleed. Awaiting spontaneous clearance of the drug is a reasonable option in these patients.

## 4. Nonspecific Reversal Agents

Several nonspecific reversal agents are used for reversal of NOAC anticoagulation. Tranexamic acid is used off-label as a hemostatic agent in major NOAC-associated bleeds [19], but currently, there is no sufficient data on its efficacy. There is an ongoing study due to be completed in December 2019 which seeks to evaluate the efficacy of tranexamic acid in patients with NOAC-ICH [20].

Prothrombin complex concentrate (PCC), is a mixture of 3 or 4 coagulation factors, and is used off-label to reverse NOACs [21–23]. Inactivated 4 factor PCC (Kcentra®) may be used in cases of factor Xa inhibitor-associated bleeding. However, activated PCC (aPCC, factor VIII inhibitor activity bypassing agent FEIBA®) is preferred for dabigatran [24, 25]. A randomized double-blinded placebo controlled study by Erenberg et al. tested the efficacy of PCC by using either a single bolus of 50 IU/kg PCC (Cofact) or a similar volume of saline in patients on rivaroxaban 20 mg twice daily (*n* = 6) or dabigatran 150 mg twice daily (*n* = 6) for 2½ days. The study included 12 male subjects (rivaroxaban, *n* = 6, and dabigatran, *n* = 6). The results from the above study suggested that the 50 IU/kg PCC immediately and completely reversed the anticoagulant effect of rivaroxaban but failed to reverse the anticoagulant action of dabigatran [25]. A recent study by Song et al. suggested that 50 IU/kg PCC was effective in reversing the anticoagulant action of apixaban as well [26]. The efficacy of PCCs on clinical outcomes on patients with NOACs and active bleeding is not yet established in a randomized control trial although observational studies are suggestive of efficacy in achieving hemostasis [27]. The European Heart Association recommends the use of PCC or aPCC in the absence of specific reversal agents in patients with major bleeding. A dose of 50 IU PCC/kg BW or 50 IU activated PCC/kg BW is recommended. No renal or hepatic dose adjustments have been reported. In the setting of NOAC-ICH, PCC does not have proven efficacy [28].

A recent study by Schulman et al. evaluated a fixed dose of PCC for the reversal of major bleeding in patients on apixaban or rivaroxaban [29]. The study included 66 patients, majority with intracranial or gastrointestinal bleeding (36 and 16 patients, respectively). A fixed PCC dose at 2,000 IU was administered, and the effectiveness of the treatment was assessed at 24 hours. Hemostasis was noted to be good in 65%, moderate in 20%, and poor/none in 15% of the study population. Nine deaths (14%) were reported at the 30-day follow-up along with five (8%) major thromboembolic events [29].

In a single-center retrospective analysis, 64 patients who presented with major bleeding were either on apixaban, dabigatran, or rivaroxaban and were given varying doses of FEIBA (factor eight inhibitor bypass activity), an activated PCC [30]. 38 patients were given low-dose FEIBA (mean 10.0 ± 3.6 units/kg) and 26 received moderate-dose (mean 24.3 ± 2.1 units/kg) FEIBA. Six patients who were on dabigatran were given idarucizumab as FEIBA was unable to adequately control severe bleeding. Four patients in the initial low-dose group subsequently received an additional FEIBA dose [30]. There was no clinically concerning active bleeding on follow-up exams after FEIBA administration except for two patients with intracranial hemorrhage who subsequently passed away. At 30-day follow-up, nine deaths (14%) and 5 thromboembolic events (8%) were reported [30].

Disappointingly, fresh frozen plasma (FFP) has shown no improvement in bleeding outcomes. It is noteworthy to mention that the benefit of blood-component therapies or prohemostatic agents in the absence of coexisting coagulopathy remains unclear [31].

Activated charcoal may reduce absorption in cases of acute over ingestion of NOACs [32–34]. It can be administered within 1-2 hours of intake to prevent NOAC absorption, but there is no evidence on its efficacy beyond 2 hours of the last NOAC dose [32, 33, 35–38]. A major concern with the use of activated charcoal is the increased risk of aspiration, especially in patients with a decreased level of consciousness [39, 40]. Preclinical studies suggest the use of hemodialysis in patients on dabigatran due to its weak affinity for plasma proteins and predominant renal excretion [41, 42]. But, hemodialysis is not suggested in patients on factor Xa inhibitors due to their high degree of plasma protein binding [43–45].

The utilization of these nonspecific reversal agents for life-threatening bleeding in patients on NOACs is expected to decrease with increasing availability of idarucizumab and andexanet alfa.

## 5. Specific Reversal Agents

### 5.1. Idarucizumab

Idarucizumab is a humanized antibody fragment with a half-life of 45 minutes. It acts by binding directly to dabigatran to counteract its anticoagulant effect. Idarucizumab is renally cleared [46] (see Table 1). To reverse the effects of dabigatran, two separate 2.5 g/50 mL vials (total of 5 g) are administered intravenously. A 2019 update of the 2014 AHA/ACC/HRS guidelines for the management of patients with atrial fibrillation recommends the use of idarucizumab for the reversal of dabigatran in life-threatening bleeding or for urgent procedures (Class I) (COR I, LOE B-NR) [47].

In healthy patients exposed to varying doses of idarucizumab to reverse dabigatran, administration of idarucizumab resulted in an immediate and complete reversal of the anticoagulant effect of dabigatran with no clinically relevant safety issues [48]. The reversal effects of idarucizumab on active dabigatran (RE-VERSE AD, NCT02104947) evaluated the efficacy of idarucizumab in patients with uncontrolled bleeding or for emergent procedure [49]. In patients presenting within 24 hours of an episode of overt bleeding, the median time to control of bleeding was 2.5 hours after idarucizumab administration. In 93% of patients who underwent surgery or intervention, the median time for periprocedural hemostasis was 1.6 hours after preprocedural idarucizumab infusion.

Idarucizumab shares structural similarities with thrombin and mimics its binding to dabigatran [46]. In phase I studies, coagulation parameters remained unchanged across a wide range of idarucizumab doses [46]. Idarucizumab was safe and effective in the reversal of dabigatran anticoagulant activity [48].

In the full cohort analysis of phase III (REVERSE-AD) study, thrombotic events occurred in 4.8% of patients (24 of 503 patients) within 30 days of treatment and 6.8% (34 patients) within 90 days [49]. None of the patients were receiving anticoagulant therapy at the time of the thrombotic event. [49]. Mortality rate was 18.8% at 90 days [49].

There are isolated case reports of idarucizumab reversing dabigatran-induced acute kidney injury [50], and idarucizumab with FFP reversing dabigatran induced diffuse alveolar hemorrhage [51].

The RE-VECTO international surveillance program enrolled 359 patients from 61 sites (Asia Pacific—13.9%, EU—42.3%, North America—43.7%) which licensed and dispensed idarucizumab [52]. Among the enrolled patients, 97.5% of patients were receiving dabigatran. 57% of the patients received idarucizumab to reverse major bleeding (gastrointestinal bleed—44.4%; intracranial bleed—38.6%), and 36% received idarucizumab for urgent interventions [52]. 95% of patients received full dose of two vials (2.5 g × 2), and only 1% received a second dose similar to findings of the RE-VERSE-AD study in which a second round dosing was required in 1.6% of patients [52]. The study suggested that the usage patterns of idarucizumab aligned with its prescribing information, and there was minimal off-label use.

The recent 2018 guidelines from the AHA and American Stroke Association provided a class III recommendation in administering IV tissue plasminogen activator (tPA) to patients on factor Xa inhibitors or direct thrombin inhibitors unless coagulation laboratory parameters are within reasonable limits or the respective NOACs have been on hold for at least 48 hours [53]. A recent systematic review found that the rate of symptomatic hemorrhage in patients on dabigatran treated with idarucizumab prior to administration of intravenous tPA was 4.5% (vs. 7.4%) and mortality rate was 4.5% (vs. 12.0%) compared to those who did not receive the reversal agent [54]. The observed rates of intracerebral hemorrhage (ICH) in patients receiving IV tPA after reversal of dabigatran was comparable to previously reported rates of ICH on patients who were not anticoagulated.

### 5.2. Andexanet Alfa

Andexanet alfa is a modified human factor Xa decoy protein that binds and sequesters factor Xa inhibitors. It has a half-life of approximately 1 hour. High-dose andexanet alfa is administered to patients receiving a factor Xa inhibitor (rivaroxaban >10 mg, apixaban >5 mg, or unknown dose) or if ≤8 hours have elapsed since its last dose. High dose consists of 800 mg IV bolus at a target rate of 30 mg/min followed by 8 mg/min continuous infusion for 120 minutes (960 mg); otherwise, a low-dose andexanet alfa is given as 400 mg IV bolus at a target rate of 30 mg/min and then a 4 mg/min continuous infusion for 120 minutes (480 mg). The AHA/ACC/HRS recommends andexanet alfa for the reversal of apixaban and rivaroxaban in patients with life-threatening or uncontrolled bleeding (class IIa) (COR IIa, LOE B-NR). Andexanet alfa has not yet received FDA approval for reversal of edoxaban, betrixaban, or enoxaparin.

Two parallel trials, the Andexanet Alfa for the Reversal of Factor Xa Inhibitor Activity (ANNEXA-A for apixaban (NCT02207725) and ANNEXA-R for rivaroxaban (NCT02220725)), studied the efficacy of andexanet alfa among healthy older volunteers [55]. The administration of either a bolus dose or one followed by a continuous infusion of andexanet alfa resulted in a rapid and significant reduction in anti-factor Xa activity as compared to placebo, with almost all participants achieving ≥80% reduction. Thrombin generation increased in 100% of participants on apixaban and 96% on rivaroxaban compared with 11% and 7%, respectively, in the placebo group. There were no serious adverse events in either group during the entire duration of the study.

The clinical trial ANNEXA-4 (NCT02329327) [56] evaluated the efficacy of andexanet alfa to achieve hemostasis for major bleeding associated with rivaroxaban, apixaban, edoxaban, or enoxaparin. In this study, the majority of subjects had a significant thrombotic disease or cardiovascular burden. There was a 92% reduction in median anti-factor Xa activity after andexanet alfa treatment in patients with acute major bleed, who received apixaban, rivaroxaban, or edoxaban within 18 hours. The clinical trial found an adequate clinical hemostasis in 85% of patients with gastrointestinal bleeding and 80% of patients with intracranial bleeding within 12 hours of andexanet alfa administration. At 30-day follow-up period, 14% of patients were deceased (49 patients) [56]. 35 died of cardiovascular causes, 12 died of noncardiovascular causes, and 2 were unknown. 10% (34 patients) reportedly had a thrombotic event myocardial infarction occurring in 7, ischemic stroke in 14, deep vein thrombosis in 13, and pulmonary embolism in 5 patients [56]. The study did not find a significant relationship between a reduction in anti-factor Xa activity and hemostatic efficacy during treatment with andexanet, except for those with intracranial hemorrhage [56]. Monitoring of anti-factor Xa activity demonstrated profound improvement immediately following completion of the bolus. While this improvement sustained through the end of the two-hour infusion protocol, however, the anti-factor Xa activity rebounded dramatically at four hours after the initiation of the bolus [56].

The use of andexanet alfa for patients on betrixaban resulted in a rapid reversal of anticoagulation; however, data is currently limited to healthy subjects [57]. An ongoing clinical trial aims at evaluating the efficacy of andexanet in patients receiving an oral factor Xa inhibitor who present with intracranial hemorrhage (NCT03661528) [58].

## 6. Laboratory Parameters

In cases of acute bleeding, emergent need for surgical intervention, need for intravenous thrombolysis, acute intracerebral bleeding, and suspected anticoagulant overdose, routine anticoagulation with assays such as prothrombin time (PT), activated partial thromboplastin time (aPTT), or thrombin time (TT) may provide limited information of drug concentration (see Table 2). In such situations, point-of-care tests (POCTs) can provide immediate information on NOAC concentration, facilitating emergent reversal. Coaguchek POCT may guide decision to thrombolyze patients on rivaroxaban presenting with ischemic stroke in the absence of availablity of anti-Xa testing [67]. Activated coagulation time-low range (ACT-LR) was tested for dabigatran using a portable Hemochron Signature Elite and prothrombin time (expressed as INR) was tested for rivaroxaban by Coaguchek XS Pro [68]. The correlation between the NOAC concentration and the obtained values using the aforementioned POCTs was high for dabigatran (*r* = 0.80 for ACT-LR) and rivaroxaban (*r* = 0.82 for Coaguchek XS Pro) but was low for apixaban [68].

aPTT and PT are readily available tests, but they lack sensitivity and specificity to adequately assess and monitor the anticoagulant effect of NOACs [69]. A liquid chromatography or tandem mass spectrometry is the most accurate method to measure drug concentrations; however, this method is not widely available. Anti-factor Xa chromogenic assays are available to measure the plasma concentrations of factor Xa inhibitors and using these assays, the absence of factor Xa activity can reliably exclude the presence of clinically significant drug levels [66].

A set of NOAC concentration values that lead to clinically significant coagulation impairment is yet to be established in a prospective trial [70]. A concentration lower than 30 ng/mL indicates the absence of clinically relevant anticoagulant activity of rivaroxaban, apixaban, and dabigatran [18, 71, 72].

Dilute thrombin time (DTT) and ecarin-based assays, with high degree of linearity with dabigatran, may quantify and monitor the activity of dabigatran. A normal aPTT can exclude dabigatran drug levels above the on-therapy range but cannot exclude dabigatran levels in the on-therapy range [66]. Thrombin time is a valuable tool to detect relevant dabigatran concentrations in blood; however, it cannot monitor dabigatran therapy [73, 74].

## 7. Resumption of NOACs 

In cases of a minor bleed, a single dose of anticoagulation is skipped or delayed.

In patients experiencing major bleeding, a careful reassessment of the risks and benefits of reinitiating anticoagulation is required [66]. Among those who present with nonmajor bleeding precipitated by an event that is reversible or unlikely to recur (e.g., unexpected trauma and bleeding from a removable mass), anticoagulation may be resumed after achieving hemostasis. In patients with a significant risk of thromboembolic disease who present with major bleeding or life-threatening bleeding, the decision to continue anticoagulation should be individualized based on risks and benefits, as well as patient preference [75].

Cessation of anticoagulation after intracranial bleeding in the setting of atrial fibrillation is associated with ischemic stroke and mortality [66, 76–78]. There are no evidence-based guidelines on the appropriate time to resume NOACs in this subgroup. Therefore, the resumption of oral anticoagulation after intracerebral hemorrhage remains a major clinical dilemma. The location of the intracerebral hemorrhage is a factor in the decision to resume anticoagulation. Lobar ICH is considered a manifestation of underlying cerebral amyloid angiopathy. Nonlobar ICH is associated with hypertensive microvascular disease. Lobar ICH demonstrates a significantly higher recurrence rate of bleeding compared to nonlobar ICH. The American Stroke Association recommends avoidance of oral anticoagulation in patients after lobar ICH and a case-by-case approach to patients with nonlobar ICH [79]. In contrast, a study by Biffi et al. found that resumption of oral anticoagulation resulted in a decreased risk of ischemic stroke after both lobar and nonlobar ICH without an increase in the recurrence of ICH [80]. A joint analysis of 3 large observational studies on ICH found that resumption of NOAC was associated with a higher likelihood of functional recovery and a lower modified Rankin scale one year post-ICH [81].

In patients on vitamin K antagonists who present with intracerebral hemorrhage, ones on concomitant antiplatelet therapy were found to have a larger volume of hematoma, increased mortality, and worse functional outcome, but not in patients on NOACs [82].

Among those with subarachnoid bleeding secondary to arteriovenous malformation or aneurysm, consider resumption of NOAC after interventional repair or surgical intervention. Also, consider a repeat brain imaging prior to restarting anticoagulation in patients with postepidural or subdural hematoma. If the repeat imaging shows stable hematoma without mass-effect or midline shift, NOACs may be resumed in 4–8 weeks after the bleeding episode, if there is no ongoing (chronic) alcohol abuse or a substantial risk of falling [66].

Gastrointestinal bleeding (GIB) is one of the most frequently encountered bleeding manifestations. Presence of various factors such as older age, previous major bleeding, concurrent heavy alcohol use, multiple angiodysplasias in the GI tract, and the need for antiplatelet therapy after percutaneous coronary intervention (PCI) are considered unfavorable for the resumption of NOACs. In addition, the risk of recurrence may outweigh the benefits of anticoagulation in patients with no clear etiology for GIB. If the net assessment favors the continuation of anticoagulation, NOACs may be initiated as early as 4–7 days after achieving hemostasis [66].

NOACs may be safely resumed within 6–8 hours in patients who undergo interventions with a minor bleeding risk, such as dental extractions, abscess incision, or superficial surgeries. In patients who undergo procedures with higher bleeding risk, NOACs may be resumed 48–72 hours postoperatively after a careful review of co-morbidities, co-medications and baseline lab work up.

## 8. Agents in Development

- Ciraparantag (aripazine or PER 977) is a synthetic molecule designed to be a universal antidote and to reverse the effects of unfractionated heparin, low-molecular-weight heparin, oral direct factor Xa inhibitor, and dabigatran [83]. The antidote binds to its target by charge-charge interactions and hydrogen bonding. It has received a fast-track designation by the FDA. The pharmacokinetic and pharmacodynamic effects of PER 977 were studied in a double-blind placebo-controlled trial [84]. Eighty healthy male subjects were given IV PER977 in escalating doses (5 to 300 mg) alone and after receiving edoxaban 60 mg (NCT01826266). Patients who received 100–300 mg showed normalization of whole blood clotting time and mean fibrin-fiber diameter within 10–30 minutes of antidote administration. No procoagulant activity was found based on the measurements of prothrombin fragment 1.2, d-dimer, tissue factor pathway inhibitor, and whole blood clotting time. The drug is currently in phase II clinical trial to study the reversal effects on apixaban (NCT03288454) and rivaroxaban (NCT03172910). Plans for phase III clinical trials were prevouly announced. The development of ciraparantag has slowed over the past few years due to difficulties in testing its effect. Ciraparantag binds to contact pathway activators such as celite, kaolin, and in vitro anticoagulants such as citrate, EDTA, and heparin. Thus, it interferes with assays that use these reagents. A point-of-care whole blood clotting time is in development for the measurement of anticoagulants in the presence of ciraparantag to overcome these limitations [85, 86].
- Zymogen-like FXa is a catalytically inactive form of factor Xa (Gla-domainless FXaS195A and GD-FXaS195A) that binds to the NOAC to counteract its effects on endogenous factor X. It is theorized to be effective in patients before the onset of bleeding by removing the inhibitor through molecular engagement but may not be useful for control after the onset of bleeding. The available data is limited to its efficacy and potency in vivo [87].
- Engineered factor Xa variants are derivatives of factor Xa. Available data shows that modification of the active site of factor Xa can disrupt apixaban binding to the active site of factor Xa. The FX(a)-C variant restores the thrombin generation in human plasma mixed with apixaban. No in vitro hypercoagulability was reported with mixing of the variant with plasma in the absence of factor Xa inhibitors [88].

## 9. Clinical Implications

An awareness of the challenges associated with NOAC-related life-threatening bleeds, updates on NOAC-specific antidotes, and nonspecific hemostatic measures is necessary for all healthcare providers prescribing NOACs. The advent of idarucizumab and andexanet alfa has offered healthcare providers a perceived notion of an enhanced safety profile. The experience with reversal agents is limted outside of trials; however, more clinical data should be available with its increased usage. In patients requiring urgent surgery or procedures, the efficacy and safety of idarucizumab is tested in clinical trials, but the efficacy and safety of andexanet alfa has not been tested yet. Idarucizumab and andexanet alfa are currently not widely available limiting their usage. Furthermore, both andexanet alfa and idarucizumab are expensive, which limits their application in clinical practice. Increased usage of reversal agents in real-life practice may enhance the safety profile of NOACs [46].

## 10. Conclusions

The approval of reversal agents, idarucizumab for dabigatran and andexanet alfa for apixaban and rivaroxaban, has provided clinicians with an option to rapidly reverse the effect of NOACs in addition to the previously available nonspecific reversal agents and possibly improve patient outcomes. Accessibility to the reversal agents and their high cost remain a challenge. A larger amount of clinical data on reversal agents should be available with their increased usage.

## Figures and Tables

**Table 1:** Available NOAC-specific Reversal agents for Non-vitamin K Antagonist Oral Anticoagulants.

	Idarucizumab	Andexanet alfa	Ciraparantag [46]
Structure	Monoclonal antibody fragment	Modified factor Xa decoy protein	Synthetic water-soluble molecule

FDA status	Approved	Approved	Under FDA review

NOACs reversed	Dabigatran	Apixaban	Apixaban^*∗*^
		Rivaroxaban	Rivaroxaban^*∗*^
			Edoxaban^*∗*^
			Dabigatran^*∗*^

Mechanism of action	Binds free and thrombin-bound dabigatran	Binds to the active site of factor Xa inhibitors	Direct binding to anticoagulants

Onset of action	10–30 minutes	2–5 minutes	10 minutes

Half-life	45 minutes	1 hour	45 minutes

Dosage form	2.5 g/50 mL solution in a single-dose vial	100 mg and 200 mg vials to be reconstituted with 10 mL or 20 mL sterile water respectively	—

Dose	5 g IV given as two-50 mL bolus infusions with 2.5 g each within 15 minutes apart	Low dose: 400 mg IV bolus at a target rate of 30 mg/min then a 4 mg/min continuous infusion for 120 minutes (480 mg)	100 to 300 mg IV one time bolus
		High Dose1: 800 mg IV bolus at a target rate of 30 mg/min then an 8 mg/min continuous infusion for 120 minutes (960 mg)	

Dose adjustment	None reported	None reported	None reported

Contraindications	None reported	None reported	None reported

Adverse reactions	REVERSE-AD: 30-day thrombotic events 4.8%	ANEXXA-4: 30-day thrombotic events 10%	Perioral and facial flushing, dysgeusia [46]
			No pro-coagulant activities reported on current clinical data
	Others:	Others:	
	Constipation 7%	Urinary tract infection >5%	
	Headache >5%	Pneumonia >5%	
	Nausea 5%	Infusion-related reactions >3%	
	Hypersensitivity reactions		

Cost	$3,482 per reversal^3^	Low dose: $29,040	—
		High dose: $58,080	
		Calculated from $3,300 per 100 mg vial^2^	

^1^Indications for high-dose and exanet alfa: *Rivaroxaban*: Last dose >10 mg or unknown AND received within <8 hours or unknown. *Apixaban*: Last dose >5 mg or unknown AND received within <8 hours or unknown. ^2^Approximate wholesale acquisition cost or manufacturer's published price. ^3^Buchheit J, Reddy P, Connors JM. Idarucizumab (Praxbind) Formulary Review. Crit Pathw Cardiol. 2016; 15 (3):77–81. ^*∗*^Based on current available data.

**Table 2:** Laboratory parameters among various NOAC's [37, 59–66].

	Apixaban [59]	Edoxaban [60, 61]	Rivaroxaban [62, 63]	Betrixaban [64]	Dabigatran [37, 65]
Prothrombin time (PT)	Linear/ curvilinear correlation	Linear, concentration-dependent, Poor sensitivity at low drug levels	Weak correlation at increasing concentrations	Concentration-dependent correlation at on-therapy range, Low sensitivity	Poor correlation, Low sensitivity

Activated partial thromboplastin time (aPTT)	Weak correlation, poor sensitivity	Fair concentration- dependent correlation	Poor to moderate concentration- dependent correlation	Concentration-dependent correlation, Low sensitivity	Degree of prolongation poorly correlated with concentration

Anti-factor Xa assay	Linear variation, decreased correlation at lower concentrations	Linear, concentration-dependent correlation	Linear, concentration- dependent correlation	Concentration-dependent correlation	—

Thrombin time	—	—	—	—	Normal TT effective in excluding significant dabigatran presence, highly sensitive

Dilute thrombin time	—	—	—	—	Strong correlation within on-therapy range

Ecarin based-assay	—	—	—	—	Strong, linear correlation within on-therapy range, highly sensitive
